# Cadmium Tolerance and Removal from *Cunninghamella elegans* Related to the Polyphosphate Metabolism

**DOI:** 10.3390/ijms14047180

**Published:** 2013-03-28

**Authors:** Marcos A. B. de Lima, Luciana de O. Franco, Patrícia M. de Souza, Aline E. do Nascimento, Carlos A. A. da Silva, Rita de C. C. Maia, Hercília M. L. Rolim, Galba M. C. Takaki

**Affiliations:** 1 Department of Biology, Federal Rural University of Pernambuco, Recife, PE 52171-900, Brazil; E-Mails: mablima33@yahoo.com.br (M.A.B.L.); lucianafranco@terra.com.br (L.O.F.); 2 Nucleus of Research in Environmental Sciences, Catholic University of Pernambuco, Recife, PE 52050-590, Brazil; E-Mails: tyttams@hotmail.com (P.M.S.); elesbao@unicap.br (A.E.N.); calves@unicap.br (C.A.A.S.); 3 Department of Veterinary Medicine, Federal Rural University of Pernambuco, Recife, PE 52171-900, Brazil; E-Mail: rccmaia@yahoo.com.br; 4 Postgraduate Program in Pharmaceutical Sciences, Federal University of Piauí, Teresina, PI 64049-550, Brazil; E-Mail: hercilia_rolim@yahoo.com.br

**Keywords:** heavy metal, cadmium, polyphosphate, tolerance, *Cunninghamella elegans*

## Abstract

The aim of the present work was to study the cadmium effects on growth, ultrastructure and polyphosphate metabolism, as well as to evaluate the metal removal and accumulation by *Cunninghamella elegans* (IFM 46109) growing in culture medium. The presence of cadmium reduced growth, and a longer lag phase was observed. However, the phosphate uptake from the culture medium increased 15% when compared to the control. Moreover, *C. elegans* removed 70%–81% of the cadmium added to the culture medium during its growth. The *C. elegans* mycelia showed a removal efficiency of 280 mg/g at a cadmium concentration of 22.10 mg/L, and the removal velocity of cadmium was 0.107 mg/h. Additionally, it was observed that cadmium induced vacuolization, the presence of electron dense deposits in vacuoles, cytoplasm and cell membranes, as well as the distinct behavior of polyphosphate fractions. The results obtained with *C. elegans* suggest that precipitation, vacuolization and polyphosphate fractions were associated to cadmium tolerance, and this species demonstrated a higher potential for bioremediation of heavy metals.

## 1. Introduction

Environmental pollution caused by toxic heavy metal due to industrial developments is one of the most significant problems of this century. In particular, cadmium is a hazardous pollutant to various ecosystems and to human health. Cadmium together with lead has been recognized as one of the major environmental and public health problems. In humans, it has been implicated as the cause of renal disturbances, lung insufficiency, bone lesions, cancer and hypertension [1,2]. Moreover, cadmium is carcinogenic, embryotoxic, teratogenic and mutagenic [3,4]. The main source of cadmium contamination is wastewater from industries of electroplating, pigments, plastic, battery and zinc refining mining [5].

Decontamination of heavy metals in the soil and water around industrial plants has been a challenge for a long time. The use of microorganisms for the recovery of metals from waste streams, as well as the employment of plants for landfill application, has achieved growing attention [6]. Lower cost and higher efficiency at low metal concentrations make biotechnological processes very attractive in comparison to physicochemical methods for heavy metal removal [7,8].

It is well established that microorganisms are able to remove heavy metals [7,9]. The capacity of metal uptake by microorganisms has attracted great attention, due to its potential to provide an effective and economic alternative when compared to conventional process of remediation of the contaminated environment with heavy metals [10]. Microorganisms able to grow in the presence of heavy metals with a significant metal uptake capacity have a potential use in bioremediation of contaminated waste residue in waters and soil [11]. Live and dead microorganisms or their derivatives can be used for removal of heavy metals ions from wastewater [12–14].

However, living, active and growing cell have the advantage of self-replenishment, continuous metabolic uptake of heavy metals after physical adsorption and the potential for optimization by genetic manipulation [9,15]. Fungi, in common with other microbial groups, can accumulate metals from their external environment. Furthermore, fungi are easy to grow, produce high yields of biomass using inexpensive growth media and, at the same time, can be manipulated genetically and morphologically [16].

On the other hand, inorganic polyphosphate (polyP) is a linear polymer of orthophosphate that has been associated to several biological functions, such as an energy source, phosphate storage, chelating of metal ions, buffering against alkali, membrane channels, enzyme and gene activity control, bacterial pathogenesis, phosphate pumps, regulation of stress, survival and development and in the formation and function of cell surface structures [17]. Moreover, studies have associated polyP to the tolerance of microorganisms to toxic heavy metals [18–21].

The resistance consists in mechanisms that inhibit or limit the toxic effects of heavy metals, while tolerance involves strategies that reduce or offset the harmful consequences of oxidative stress [22].

Therefore, polyP is a polyfunctional biopolymer, and its functions depend on the type of organism, cellular localization, the amount and when it is needed [17]. According to its solubility in acid and alkali, polyP can be separated into distinct fractions, which differ in both molecular weight and localization and functions in the cell. Studies have shown that the content and chain length of different polyP fractions depends on the growth phase of the lifecycle, as well as on the culture conditions [23].

Fungus species, *Cunninghamella elegans*, has great ability to accumulate phosphorus in the form of polyphosphate [24], to metabolize xenobiotic recalcitrant substances, such as polycyclic aromatic hydrocarbons-PAH [25], azo dyes used in the textile industry [26], oxidation of dibenzothiophene [27] and biotransformation of drugs [28], as well as it is able to grow in the presence of copper [29].

However, there is a lack of information on the metal ion removal ability of *Cunninghamella elegans* and on the role of polyP fractions in the tolerance to heavy metals in the literature. Thus, this investigation reports studies on the capacity of *C. elegans* growth and cadmium removal from culture associated to polyphosphate metabolism.

## 2. Results and Discussion

### 2.1. Effect of Cadmium in the Growth and Phosphate Consumption of *C. elegans*

**Figure 1:**
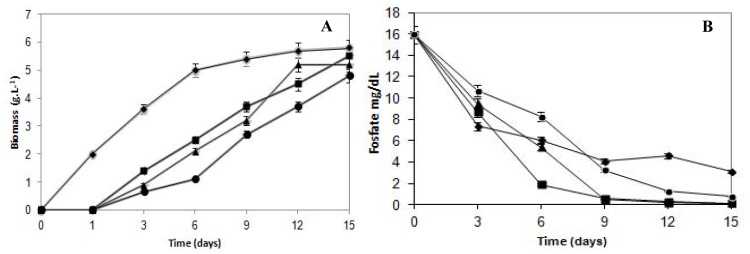
Growth and phosphate consumption curves of *C. elegans* in cadmium-containing synthetic medium for Mucorales (SMM) medium during 15 days at 28 °C and orbital shaking of 150 rpm. Growth (**A**) and phosphate consumption (**B**) curves. Samples: untreated control (-♦**-**) and treated with cadmium concentrations of 5.62 mg/L (-■-), 11.24 mg/L (-▲ -) and 22.10 mg/L (-●-).

The effects of cadmium on the growth of *C. elegans* are presented in Figure 1A. The fungal growth pattern of the untreated control sample exhibited typical growth phases. On the other hand, cultures submitted to cadmium treatment presented a continuous growth during the assay period. The growth in the presence of cadmium concentrations (5.62 mg/L, 11.24 mg/L and 22.10 mg/L) increased the lag phase, and a one day long lag phase was observed compared with the control sample.

Cadmium altered the normal growth profile of the bacteria and fungi growing in the culture medium [30–32]. Donmez and Aksu [33] reported elevation of the lag phase during growth of *Candida* sp. in the presence of copper and nickel and *Trichoderma viride* in copper [34].

Fungal biomass decreased in a proportional way in response to the metal presence in the culture medium. The Fungal growth in the media with cadmium compared with control culture showed the highest reduction during the initial six days of incubation. In this stage, the growth was reduced 51%, 59% and 78% to cells cultivated in cadmium content of 5.62 mg/L, 11.24 mg/L and 22.10 mg/L, respectively. Subsequently, when establishing the stationary growth phase of the control culture, the difference in the biomass production was progressively diminished, when compared with samples grown in the presence of cadmium.

The final biomass production was 5.8 g/L for control and 5.5, 5.6 and 4.8 g/L when submitted to 5.62 mg/L, 11.24 mg/L and 22.10 mg/L of cadmium, respectively. Thus, cadmium concentrations limited, but did not inhibit, the growth of *C. elegans*. Results indicated that *C. elegans* can tolerate cadmium up to a concentration of 22.10 mg/L.

In this work, the biomass production of *C. elegans* in the presence of cadmium was similar or even superior to the biomass production of the majority of fungi reported by Massaccesi *et al.*[35] grown in a concentration of 10 mg/L of cadmium, suggesting that *C. elegans* may be acquiring physiological adaptation to this heavy metal.

Regarding the phosphorus consumption curves (Figure 1B), it can be observed that up to the third day of culture, the control samples consumed more phosphorus than those treated with metal at any concentration. However, after the sixth day of incubation, when the growth curves started showing a remarkable increase, the cultures cultivated with cadmium started consuming more phosphorus than the control mycelium. Therefore, at the end of the growth, the total phosphorus consumption by the control samples reached 80%, whereas cadmium-treated cultures at 5.62 mg/L, 11.24 mg/L and 22.10 mg/L reached 99%, 99% and 95%, respectively.

It is important to report that although the biomass production was 53% lower than the control samples, the 22.10 mg/L cadmium-treated cultures, after nine days of growth, showed phosphorus consumption 17% higher than the control. Therefore, it is evident that the culture cells’ phosphorus requirement in the presence of cadmium was much higher than the cells from the control group, demonstrating that the phosphorus levels consumed by cadmium-treated cells that presented lower growth rates exceed the normal physiological needs. This finding suggests that the phosphorus supply is being used to support the fungal survival in the presence of the heavy metal, and for that reason, it is possible to infer that phosphorus is involved in the cadmium detoxification process in *C. elegans*.

Aiking *et al.*[36] report that the phosphate accumulation by *Klebsiella aerogenes* during growth in a cadmium-containing medium could be considered a primary mechanism of detoxification, since it causes the formation of cadmium phosphate, an almost insoluble salt that can precipitate, reducing the metal toxicity.

### 2.2. Removal Characteristics of Cadmium by *C. elegans*

Experiments were carried out to study the effect of varying initial cadmium concentration on cadmium removal of culture medium by *C. elegans* (Table 1). The isotherm equilibrium was not determined here, because fungal growth with cadmium uptake was not a restricted surface phenomenon.

The results demonstrated that metal removal biosorption capacity increased with the increase in initial cadmium concentration. The highest removal efficiency of cadmium, 81%, was reached when using the lowest concentration (5.62 mg/L), and the cadmium removal efficiency was 40 mg/g with the velocity of cadmium removal of 0.031 mg/h. When *C. elegans* was grown on 11.24 mg/L of cadmium, it showed a biosorption capacity of 90 mg/g, and the velocity of cadmium removal was 0.061 mg/h. However, the treatment with 22.10 mg/L of cadmium showed a biosorption capacity of 280 mg/g, and the velocity of the cadmium removal was 0.107 mg/h.

Furthermore, it can be observed that the removal velocity was dependent on the initial metal concentration, varying proportionally with the cadmium content.

Malik [15] in a review reported that the maximum uptake capacity and removal efficiency for cadmium ions from fungi was 184 mg/g by *Gliocladium roseum* species and 70% by *Talaromyces helices*, respectively. In addition, Kapoor and Viraraghavan [16] demonstrated values of maximum cadmium removal that ranged between 0.4 and 71 mg/g by various fungal species. Thus, the cadmium uptake by *C. elegans* mycelia obtained in this study when compared to the literature could be considered promising.

On the other hand, cadmium accumulation within fungal cell increased with the external metal concentrations. The highest cadmium accumulation by *C. elegans* of 212 mg/g was obtained in cells treated with 22.10 mg/L of cadmium at six days. This value corresponded to 75% of the biosorbed cadmium, indicating that the main process of metal removal was bioaccumulation. This fact can be involved in the tolerance enhancement to metal; the intracellular accumulation is a way to detoxify heavy metals.

*C. elegans* exhibit the ability to accumulate cadmium ions when metabolically active. Since living cells can present all types of non-metabolic interactions with metals, as well as interactions that require active metabolism, and based on the findings from this study, we may assume that the fungus may accumulate more metals when they are metabolically active. Malik [15] reported that growing cells have various advantages in employment for metal remediation than inactive cells.

A similar cadmium accumulation pattern has been reported by Nishikawa *et al.*[37] using the microalgae, *Chlamydomonas acidophila*. However, the uptake rate was lower when compared to the data obtained in this work with *C. elegans*.

### 2.3. Effect of Cadmium on the Hyphal Ultrastructure

**Figure 2:**
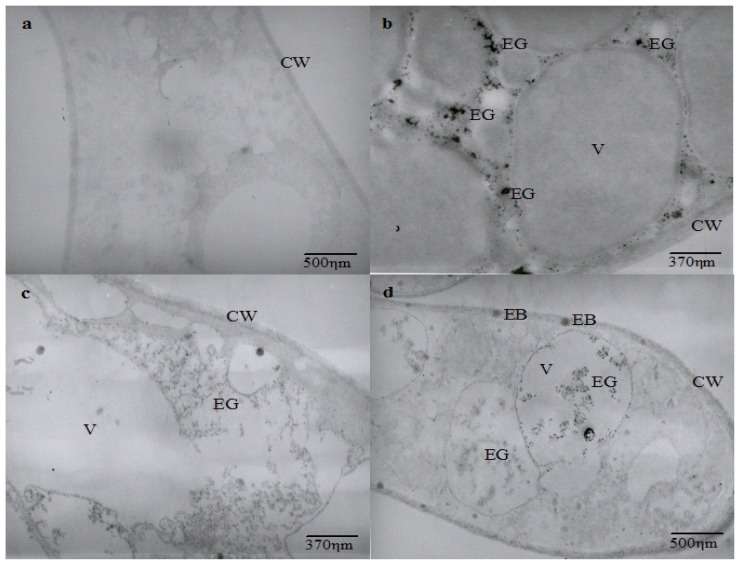
Transmission electron micrographs of *Cunninghamella elegans* cultivated in SMM medium without or with cadmium during three days at 28 °C and orbital shaking of 150 rpm. Untreated hyphae (**a**); hyphae treated with 5.62 mg/L of cadmium (**b**); 11.24 mg/L (**c**); and 22.10 mg/L (**d**). Abbreviations: CW = Cell wall; V = Vacuoles; EG = Electron-dense granulations; EB = Electron-dense bodies.

The ultrastructural analysis shows an intense vacuolization in cadmium-challenged hyphae, the presence of electron dense granulations around and inside vacuoles, as well as electron dense bodies in the cell membrane and wall that are not seen in control micrographs (Figure 2).

Ultrastructural similar changes were reported in *Chlamydomonas acidophila* cells treated with cadmium, which exhibited an increase in the number and volume of vacuoles, as well as the presence of electron dense deposits inside vacuoles and membranes. It is proposed that vacuolization represents a compartmentalization mechanism of toxic metals [37].

Cunningham *et al.*[38] also related that *Clostridium thermoaceticum* exhibited an intense distribution of electron dense precipitate during growth in the presence of cadmium and proposed this as a cellular mechanism to mediate the tolerance to the metal. The presence of electron-dense granules and bodies in different regions of cadmium-treated hyphae could be related to cadmium precipitation. However, it is necessary to determine the elementary composition of electron dense granules and bodies to demonstrate that *C. elegans* is able to use this mechanism as a process for cadmium tolerance.

In this work, the cadmium influence on the cellular growth of *C. elegans* was evaluated for the first time. Also, the cellular ability to tolerate, remove and accumulate cadmium pointed out the potential of this filamentous fungal as a biosystem to metal bioremediation.

### 2.4. Polyphosphate Levels in Hyphae Growth on the Presence of Cadmium

**Figure 3:**
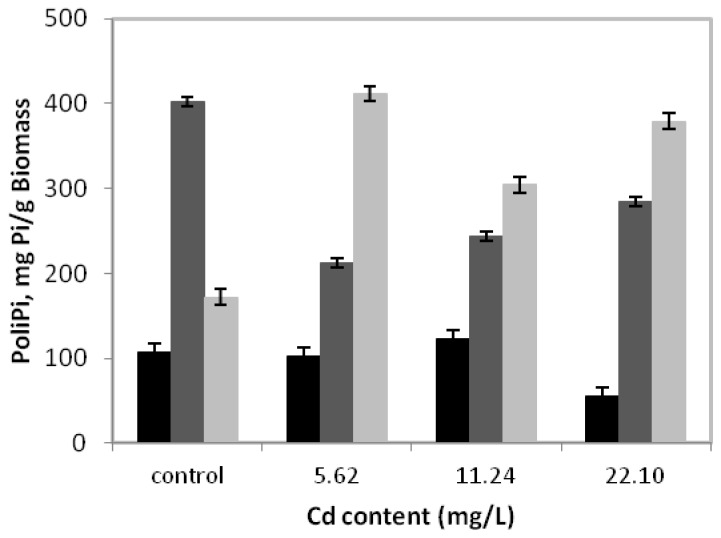
Polyphosphate fractions of *C. elegans* grown for 15 days at 28 °C and 250 rpm in SMM medium containing 5.62 mg/L, 11.24 mg/L and 20.10 mg/L of cadmium. Fractions: acid soluble (black bar), alkali soluble (dark gray bar) and acid insoluble (light gray bar).

Figure 3 presents the behavior of polyphosphate fractions during *C. elegans* growth in a medium without or with cadmium for 15 days.

It can be observed that in the control sample, the alkali-soluble fraction had the highest concentration, with a peak of 402.2 mgPi/g of biomass. Nevertheless, during growth in the presence of 5.62 mg/L of cadmium, degradation of the alkali-soluble fraction was observed, whose maximum value was 47.2% lower than that obtained in the absence of metal. However, we emphasize that in this condition, the acid-insoluble fraction levels increased significantly, reaching a peak of 411.6 mgPi/g of biomass or 239.2% higher than in the absence of cadmium. At the same time, the acid-soluble fraction was stable and continued to exhibit lower values than alkali-soluble and acid insoluble fractions.

On the other hand, the cadmium concentrations of 11.24 mg/L and 22.10 mg/L can indicate that the behavior profile of the three fractions of polyphosphate are similar when compared to the concentration 5.62 mg/L, with emphasis on the predominance of the acid-insoluble fraction.

Shari’a *et al.*[39] demonstrated the presence of polyphosphate on the cell surface, cell membrane, vacuoles and cytoplasm from three species of Zygomycetes fungi. Moreover, Lima *et al.*[24] detected polyphosphate in the form of electron-dense granules in different cellular compartments of *C. elegans*, such as cell walls, vacuoles, cell membrane and cytoplasm.

While physiological functions of the polyphosphate are not fully substantiated, this polymer has recently been associated with microorganism tolerance to heavy metals. It has been suggested that microorganisms use the polyphosphate to detoxify heavy metals [18,40].

We demonstrate for the first time the behavior of the different fractions of polyphosphate in *C. elegans* in a cadmium-containing medium. The results presented here indicate that a possible role in metal tolerance is not a major characteristic for all fractions of the polyphosphate.

There is no consensus in the literature about how the polyphosphate plays a role in heavy metal tolerance. Initially, due to the ability to polyphosphate chelate metals, it has been proposed that the ability to accumulate large quantities of this polymer would be sufficient to reduce the toxicity of the metal and, thereby, determine the resistance of a microorganism [41]. Pan Hou *et al.*[40] obtained results that confirm this hypothesis.

However, experimental evidence raised two new hypotheses to try to explain the relationship between the polyphosphate with metal resistance. The first states that the ability to degrade polyphosphate to orthophosphate is as important for metal tolerance as the ability to store it. Lima *et al.*[42] observed a drastic reduction in the content of polyphosphate during growth of *T. harzianum* in medium with cadmium. On the other hand, it has also been proposed that the ability to synthesize and degrade are equally important in the process of metal tolerance [19–21].

Our results showed a clear change of polyphosphate alkali-soluble and acid insoluble fractions, due to the presence of cadmium. Regarding the polyphosphate alkali-soluble fraction, degradation was found, while the acid-insoluble fraction accumulation was observed when cadmium was present in culture medium. Nevertheless, more studies are required to clarify the relative importance of each fraction of polyphosphate on the fungal tolerance to heavy metals.

## 3. Experimental Section

### 3.1. Fungal Strain and Growth Conditions

The strain IFM 46109 of *Cunninghamella elegans*, kindly supplied by the Culture Collection of the Research Center for Pathogenic Fungi and Microbial Toxicosis, Chiba University, Japan, was maintained in the Culture Collection UCP/WFCC (Catholic University of Pernambuco) and registered in the World Federation for Culture Collection. The strain was maintained on PDA (DIFCO) slants, incubated at 5 °C and transferred to fresh PDA slants at six month intervals. The PDA medium also was used for large scale spore production at 28 °C, during 5 days. A total amount of 10^5^ spores/mL of *C. elegans* were collected from PDA and transferred to 125 mL Erlenmeyer flasks containing 50 mL of synthetic medium for Mucorales (SMM), described by Hesseltine and Anderson [43], added with monohydrate chloride salts of cadmium containing 5.62 mg/L, 11.24 mg/L and 22.10 mg/L of cadmium. The control cultures were grown in the original medium, without cadmium. The flasks were incubated during 15 days, at 28 °C, at 250 rpm. All experiments were carried out using three replicates. The cell viability was evaluated after the incubation time by inoculating cells collected in SMM solid medium, in which the colony growth was observed.

### 3.2. Growth Curves

Biomass collected by filtration at 1, 3, 6, 9, 12 and 15 days of growth was washed twice in PBS, submitted to liophylization and maintained in a vacuum desiccator until constant weight. The final value obtained corresponds to the arithmetic media of three replicates of each biomass sample.

### 3.3. Phosphate Consumption

Samples of culture supernatant collected by filtration at 3, 6, 9, 12 and 15 days of cultivation were submitted to phosphate determination by the method of Fisck and Subbaro [44] made in a Spectronic Genesys 2. A standard curve was produced using a potassium phosphate solution (0.5 to 5.0 g/L). The final value corresponded to the arithmetic media of three replicates of each sample.

### 3.4. Removal Efficiency of Cadmium

To evaluate cadmium removal by mycelia of *C. elegans*, samples of culture supernatant were collected by filtration at 6 days or 144 h of growth. The metal concentrations were determined by atomic absorption spectrophotometry (model GBC 906), using a specific lamp and a wavelength specific for cadmium. A standard curve was produced. Experiments were conducted in triplicate, and average values were used in the analysis. The biosorption capacity *q* (mg g^−1^) and removal efficiency *E* (%) of cadmium ions by *C. elegans* was calculated according to Volesky [45] using the following equations:

(1) $$q=\frac{(C_{\text{i}}-C_{\text{f}})\times V}{Wg}$$

(2) $$E\;(\%)=\frac{C_{\text{i}}-C_{\text{f}}}{C_{\text{i}}}\times 100$$

where *C*_i_ and *C*_f_ are the initial and final metal ion concentrations (mg/L), respectively, *V* (L) is the medium volume containing heavy metal and *W* is the dry weight biomass (g). The removal velocity, RV (mg/h), was determinated by the ratio between removal cadmium and the time of cultivation.

### 3.5. Cellular Cadmium Uptake

The biomass collected at 6 days or 144 h of incubation by filtration was washed three times with 0.1 M ethylenediamine tetra acetic acid (EDTA), for 10 min, for removal of cadmium linked to cell surfaces. After this period, the biomass was dried and digested with an acidic solution constituted by concentrated HNO_3_ and HClO_4_ (9:1), for 6 h, at 150 °C. Then, the digested material obtained was diluted with 1 N HCl and analyzed for cadmium content by atomic absorption spectrophotometry (model GBC 906), and the values obtained were related to intracellular cadmium. The experiments were performed in triplicate, and average values were used in the analysis.

### 3.6. Electron Microscopy

The biomass of *C. elegans* collected after 3 days of culture were washed twice in PBS (phosphate-buffered saline) and submitted to a routine technique. Samples were fixed in 2.5% glutaraldehyde (Sigma, St. Louis, MO, USA), in 0.1M cacodylate buffer (Sigma, St. Louis, MO, USA), pH 7.4, for 1 h. After this period, it was post-fixed in 1% osmium tetroxide (Sigma, St. Louis, MO, USA) for 1 h, followed by washing in 0.1 M cacodylate buffer, pH 7.4, and dehydrated in acetone and embedded in Epon 812 (SPI-Chem, West Chester, PA, USA). Ultrathin sections 50–70 nm thick were cut with a diamond knife in a REICHERT JUNG ultramicrotome and collected on copper grids. They were stained with uranyl acetate and lead citrate and observed and photographed using a JEOL CX-100 transmission electron microscope, operating at 60 kV.

### 3.7. Extraction and Determination of Polyphosphate Fractions

Biomass collected after 15 days of growth were washed twice in PBS and submitted to sequential polyP extraction by using the method described by Smirnov *et al.*[46]. The biomass was extracted initially in 5 mL of 0.5 N HClO_4_ at 0 °C for 3 h by stirring. The resulting homogenate was centrifuged at 12,000*g* for 5 min to remove cell debris. The supernatant was the acid-soluble fraction of polyP. The precipitate was then extracted by 0.05 N NaOH (pH 12) at 0 °C for 3 h, and an alkali-soluble fraction of polyP was then obtained by centrifugation at 12,000*g* for 5 min. The remaining precipitate was again treated with 0.5 N HClO_4_ for 3 h, now at 90 °C, and an acid-insoluble fraction of polyP was obtained by centrifugation at 12,000*g* for 5 min. The orthophosphate and labile phosphorus content were determined in the acid-soluble, alkali-soluble fractions. The amount of labile phosphorus, considered to be polyP, was evaluated by the difference in orthophosphate (Pi) content prior and after hydrolysis in concentrated HCl at 100 °C for 45 min, according to the method of Macgrath and Quinn [47], based on the phosphate liberated, and was determined in a Spectronic Genesys 2.

## 4. Conclusions

Cadmium was able to induce variations on the cellular growth profile of *C. elegans* related to its concentration. Active growing cells of *C. elegans* exhibited higher cadmium removal efficiency and biosorption capacity. Moreover, the results of this study showed that the cells of this fungus can accumulate the majority of the sorbed cadmium. Alternatively, it is possible that *C. elegans* may be using the processes of precipitation, vacuolization and accumulation/degradation of polyphosphate as a detoxification mechanism.

## Figures and Tables

**Table 1:** Removal efficiency and intracellular accumulation of cadmium by *C. elegans* growing in SMM medium containing 5.62 mg/L, 11.24 mg/L and 22.10 mg/L of cadmium during 144 h at 28 °C and orbital shaking of 150 rpm.

Cd initial (mg/L)	Cadmium			

	*q* (mg/g)	*E* (%)	RV (mg/h)	Accumulation mg/g
5.62	40	81	0.031	48
11.24	90	78	0.061	77
22.1	280	70	0.107	212
